# Cases from the Surgical Clinic of the Jefferson Medical College

**Published:** 1860-01

**Authors:** Emil Fischer

**Affiliations:** Philadelphia


### Original Communications.

### ART. I.—*Cases from the Surgical Clinic of the Jefferson Medical College.*—Service of Professor GROSS.

Reported by EMIL FISCHER, M.D., of Philadelphia.

I. *Case of Congenital Cataract; Operation by Division.*—J. C., a healthy, well-developed male child, five months old, was brought to the college clinic in the early part of November, 1859, on account of congenital blindness. The patient had enjoyed good health up to this time, and there seemed to be no tendency to hereditary disease in the family.

On subjecting the eyes of the child to a careful examination, the pupil of each was observed to be obscured by a cataract. The opake body, in both eyes, was of a bluish-white color, without presenting the striated appearance so frequently observed in acquired cataract, and made the impression as if being composed of a paste-like substance; its size did not seem to exceed that of the lens in the normal state. The opacity involved both the crystalline lens and its capsule, constituting thus a capsulo-lenticular cataract; from its appearance as well as from the nature of the case, it was presumed to be of the soft kind. The iris was of a bluish-green color, and was apparently in a normal condition; the pupil obeyed the light but imperfectly.

As is apt to be the case in congenital cataract, the eyes were affected with perpetual oscillation—a circumstance which rendered the subsequent operation one of considerable difficulty.

*Operation.*—The patient being an infant at the breast, no preparatory treatment was necessary. In order to dilate the pupil, a solution of one-eighth of a grain of atropia in two drachms of water was dropped into the eyes; the dilation was, however, incomplete and irregular in consequence of adhesions between the circumference of the capsule and the iris. The child was rendered motionless by being wrapped up in a shawl firmly pinned. An assistant being seated in a chair, took it in his lap and secured it in a half-sitting posture, while another, standing behind the chair, fixed the head and the upper eyelid.

Professor Gross, sitting before the patient, and confining the lower extremities of the same between his thighs, introduced a most delicate, perfectly straight, and sharp-pointed needle two lines behind the cornea, a little below the horizontal diameter of the eye; and, directing the point of the needle in front of the lens and its capsule, lacerated and divided these structures freely, separating the capsule from the adhesions with the iris, and pushing as many fragments as possible forward into the anterior chamber. The other eye was then operated upon in a similar manner.

In order to prevent constitutional irritation, a few drops of laudanum were administered to the patient immediately after the operation, and a dose of castor-oil was ordered to be given the next morning.

The child was confined in a dark room, and had its eyes covered by a light bandage. The pupils were kept dilated until inflammatory action had subsided. Any further medical interference was considered unnecessary, and the child was put to the mother's breast as often as usual.

The operation was not followed by any untoward symptoms; the reaction subsequent to it was very slight, and the local inflammation of but little account. One week after the operation the pupil of the left eye was found to be perfectly clear; in that of the right eye, however, but little change had taken place; and in order to render the cure complete, the operation had to be repeated on the unchanged eye.

*Remarks.*—In regard to the *cause* of congenital cataract, the opinion of pathologists, said Professor Gross, is divided. Some, supposing the lens to be opake at a certain period of life, ascribe the affection to an arrest of development; others believe it to be a product of inflammation. It is highly probable that congenital cataract may originate from either cause; for, while in some cases it is accompanied by other congenital malformations, such as iridermia, coloboma of the iris, or micropthalmos, we find in others various products of inflammation coexisting with it, such as opacity of the cornea, and adhesions between the iris and the capsule.

Congenital cataract may, like acquired cataract, assume various *forms;* it may be either lenticular, capsular, or, as is most frequently the case, capsulo-lenticular. According to the opinion of several authors, the different forms seem to be, in many instances, but different stages of transformation.

Mackenzie believes that congenital cataract is generally lenticular at birth, and that only after some months it becomes capsulo-lenticular. The capsulo-lenticular cataract seems to be capable of being transformed into the simple membranous or capsular variety, the substance of the crystalline lens being gradually absorbed, while the capsule is left in an opake state. From the experience of Mr. Saunders, this change appears to be by no means uncommon, and seems to occur principally in children of a more advanced age and in adults, the disease having made already greater progress in these cases.

A peculiarity commonly noticed in cases of congenital cataract is the incessant, irregular, and rapid rolling or oscillation of the eyes; it is explainable by the circumstances that the eyes not being attracted by external objects, volition is not exercised over the muscles of these organs; in some instances, however, this irregular motion seems to be independent of the existence of cataract, and is then more probably owing to a coincident disease, as nystagmus, or clonic spasms of the motor muscles.

Congenital cataract should be operated upon as soon as possible; not only on account of the injurious influence the blindness exercises upon the education and mental development of the child, but also because, if the operation is delayed, the habit of rolling the eyes becomes more and more inveterate, and less controllable by will, the capsule acquires greater toughness, and the sensibility of the eye is lessened with advancing age.

Professor Gross does not hesitate to operate at any period, provided the eye and general system are in a sound condition. He has repeatedly operated upon children under six months, and once upon an infant hardly four weeks old, and in almost every instance with the most gratifying results. Except in a solitary instance, and then he did not have charge of the after-treatment, he has never seen anything like active inflammation after the operation, however early performed. According to his experience, children in general bear this kind of interference much better than grown persons, their nervous system, although easily shocked, recovering much sooner from the effects of the operation than that of adults.

In accordance with the rule laid down by Guthrie, that hard cataract admits only of extraction or displacement, the soft seldom of displacement or of extraction, but usually of division, the proper operation for congenital cases, in which the cataract usually occurs in the soft state, is that of division; this operation is, moreover, best adapted to the cataract of infants and young children, and to cases in which the eyes and the patient are very restless. Of the two methods of performing the operation of division, that of scleroticonyxis, or the posterior operation, deserves the preference. The operation of keratonyxis, or the anterior operation, as advocated by Saunders and others, is not only awkward of execution, especially on restless eyes, but in its performance the iris is more easily injured, adhesions cannot be so easily separated, and the fragments of the cataract are dislodged with greater difficulty.

The most embarrassing circumstances which the operator has to contend with in cases of congenital cataract are the oscillation of the eyes, mentioned above, and the toughness of the capsule of the lens; the latter makes division in many cases unusually difficult, favors the closure of the incisions in the capsule, and, interfering thereby with the quick absorption of the cataract, renders a repetition of the operation necessary.

II. *Cystic Disease of the Testicle, complicated with Scrotal Hernia; Excision; Cure.*—A. S., a German laborer, forty-eight years of age, presented himself at the clinic of Professor Gross, in October, 1859, on account of a large tumor of the right testicle. The man, who was a widower, and the father of four children, had enjoyed very good health. The tumor had existed from his earliest recollection, but had grown very slowly; it had now attained an enormous size, weighing from four to five pounds, and annoyed the patient considerably by its bulk, and a dragging pain in the loins, which was only relieved when the testicle was supported; its surface was smooth, and the skin of the scrotum unchanged in appearance, except that its superficial veins were somewhat enlarged, and rather tortuous. It was but little painful on pressure. Some fluctuation could be felt in its upper portion, but the greater part of it conveyed to the touch the sensation of a solid and elastic body. Its shape was oval, with a somewhat narrow, constricted appearance in the middle. The cord seemed to be in a healthy condition; and there was no enlargement of the glands in the groin.

In consideration of these circumstances, the history of the case, and the healthy look of the patient, the idea of the tumor being of a malignant character could be dismissed without hesitation. From simple hydrocele it distinguished itself by its shape, which was not pyriform, as in the former affection, by its greater heaviness, the absence of distinct fluctuation, by strong compression of the tumor, not causing the sickening pain usually experienced in hydrocele, and by the absence of transparency, which, however, could be ascribed to the thickened state of the sac. In order to render the diagnosis more satisfactory, an exploring needle was introduced into several parts of the tumor. The puncture of the upper portion was followed by the free escape of serous fluid, tinged with blood; the lower portion was found to be solid. It was supposed that the fluid was contained either in an old hydrocele coexisting with the disease of the testis, or in a distinct cyst. A large hernia existed in the right groin, for which the man had long worn a truss.

*Operation.*—The patient having been put under the influence of ether, a trocar was introduced into the upper part of the tumor, to empty it of its fluid contents. About twenty ounces of a dark and turbid-looking grumous fluid escaped, containing a considerable quantity of cholesterine in the form of innumerable shining particles. The tumor was thus reduced to about two-thirds of its former size, and consisted now solely of the diseased testis, the removal of which was the object of the second step of the operation.

In proceeding to this part, Professor Gross made two curvilinear incisions from the upper part of the tumor to the bottom of the scrotum, along its anterior surface, so as to embrace an elliptical piece of integument which, making proper allowance for shrinkage, would otherwise have been superabundant.

The diseased testicle was then detached from its connection with the scrotum by a rapid dissection, an assistant drawing aside the penis and the opposite gland, so as to prevent their being wounded. The spermatic cord was carefully separated from the surrounding parts, seized with a forceps, which compressed it and prevented its retraction, and cut off just above the tumor. Although the vessels of the spermatic cord were found enormously enlarged, there was no disposition to bleeding. A ligature was passed around the spermatic artery, but not a single scrotal vessel required ligation. The wound was not closed for five hours after the operation, cold water dressing being in the mean time applied to its surface. As soon as reaction took place, and the flaps relaxed, some small arteries of the scrotum commenced bleeding, and were promptly secured. The edges of the wound were then brought together by silver sutures, and dressed in the usual manner.

*Progress of the Case.*—In the succeeding night some arterial hemorrhage occurred, on account of which some of the sutures had to be opened, in order to tie the bleeding vessels. On the second day after the operation erysipelas commenced to show itself in the edges of the wound, and spread gradually over the surrounding parts, but subsided in the course of a few days, under the use of appropriate applications. Three weeks after the operation the wound had completely cicatrized, and the inflammation had caused the hernial sac to contract to so great an extent that the bowel did not descend below the external abdominal ring, in this way effecting a cure. To guard against accident, however, the patient was advised to wear a truss.

*Examination of the Tumor.*—On examining the morbid mass, after its removal, it was found to be a *cystic* tumor of the testicle, or what is commonly called a *cystic sarcoma*, and what has been described by Sir Astley Cooper under the name of *hydatid*, or *encysted disease of the testicle.* It consisted of a large multitude of cysts, imbedded in a dense, solid fibro-areolar tissue; the size of the cysts varied from that of a millet-seed to that of a hazel-nut. The younger cysts were composed of a smooth, vascular membrane, containing a thin, serous fluid; the coats of the older and larger ones were thickened and corrugated, and some of them contained a thick, glairy fluid, while others were filled with a more solid substance resembling suet, and consisting chiefly of modified epithelial cells. Microscopic examination proved the absence of cancer cells, and, consequently, the non-malignant character of the disease.

*Remarks.*—With regard to the nature and origin of the cysts in cystic sarcoma, Professor Gross does not concur in the opinion of Sir Astley Cooper, Curling, and others, that they take their rise in an obstruction and morbid dilatation of the seminiferous ducts. From an examination of the tumors in the few cases which have come under his notice, he has been led to conclude that the tubular structure of the organ had nothing to do with the development of the cysts. It is certain that hundreds, and sometimes thousands, of cysts are formed in the testicle long after its seminal tubes are apparently completely annihilated, as is proved by the large size which such tumors often attain, and by the total absence of the primitive structures. He believes, therefore, that the more plausible conjecture is that the development is effected in the plastic matter which accompanies the morbid action, in the same manner that cells are formed in the original tissues; and he supposes that the cysts found in osteo-sarcomatous formations of the lower jaw and other parts of the body have a similar origin.

III. *Operation for Traumatic Aneurism of the Anterior Tibial Artery; Ligation of the Femoral, and of the Popliteal; Recovery.*— Charles Buxenstein, thirty-five years old, a German by birth, was admitted to the clinic of the Jefferson Medical College, in October, 1859. The patient had served in the army of the United States for nineteen years, and had accompanied Colonel Fremont in his exploring expedition across the continent. He bore the signs of many wounds and injuries about him which he had received in the service, suffering from an old dislocation of the humerus on the right side, from paralysis of the left leg and arm, and from a ball imbedded in the left iliac fossa near the crest of the ilium, in addition to the injury for the relief of which he applied.

On examining his right leg a cicatrice presented itself on the external side, about three inches and a half below the head of the fibula. He had been wounded in this place about four years ago by an Indian arrow, pointed with a locust thorn. On removing the arrow from the wound a jet of arterial blood followed, which was finally stopped by compression. The wound healed; but some time afterwards a pulsating tumor developed itself in its neighborhood; the beating continued until a few months ago, when it became gradually imperceptible. The patient suffered severely whenever he exercised or fatigued himself, and found the motion of the affected limb much embarrassed; he, therefore, desired relief by an operation.

The tumor was of oval shape, a little larger than a goose's egg, and felt very solid; it was situated on the outside of the limb, in the region of the cicatrice, between the tibia and fibula. Professor Gross assumed it to be an aneurism of the anterior tibial artery, but thought it possible that the posterior tibial might be involved, although the tumor projected hardly far enough backward to make such an implication appear very probable. The hardness of the aneurism was ascribed to its being filled with a clot. If the anterior tibial artery was the injured vessel, as it seemed most likely, the wound had been received a short distance below its origin, where the vessel is deeply seated, and ligation of it is difficult to perform.

Some time before the patient applied at the clinic of the college, he had consulted a surgeon in a neighboring city, who advised amputation, a measure to which he objected, principally on account of the condition of his arms, which would have made the use of crutches impossible.

The operation proposed by Professor Gross was to cut down over the anterior tibial artery, open the aneurismal sac, turn out the clot, and ligate both ends of the artery.

*Operation.*—A tourniquet was applied over the femoral artery, and the patient being fully under the influence of chloroform, an incision, about four inches and a half in length, was made over the tumor, in the direction of the course of the anterior tibial artery. The skin, and the tissues covering the aneurismal sac having been carefully divided, the sac was opened, and an ovoidal, solid clot removed, of the size of a hen's egg; on the cardiac end of the clot a cup-shaped excavation was found, covered with soft coagulum, and evidently owing to the blood of the artery having played against that end. From the deep situation of the artery, on the interosseous ligament, it was found impossible to apply a ligature, and the hemorrhage becoming alarming, ligation of the femoral artery was decided upon. An incision was accordingly made, about three inches in length, in the situation generally chosen, along the inner border of the sartorius, so as to expose the artery in the superior part of its course; the aponeurosis and sheath of the vessel having been carefully divided, the artery was separated from the accompanying vein in the usual manner, and a ligature passed around it. In consequence of this operation the hemorrhage was decidedly diminished, but by no means entirely arrested, and there was considerable arterial bleeding from the bottom of the wound, apparently of a recurrent nature. The operator found it, therefore, necessary to tie the popliteal artery in its lower portion. An incision of about four inches having been made between the heads of the gastrocnemius muscle, the structures of the ham were separated under the precautions usually taken; the artery was finally exposed, separated from the accompanying vein, and a ligature cast around it. This measure arrested the hemorrhage completely.

*Progress of the Case.*—The patient having been carried to bed after the operation, the limb was placed in an easy and relaxed position. As its temperature was considerably reduced, and the circulation in it but feeble, the parts were wrapped up in wadding. A full dose of morphia was administered immediately after the operation, to tranquilize the action of the heart and allay pain. In the succeeding night, about ten hours after the operation, the limb became warm, the circulation slowly re-establishing itself. During the first few days the traumatic fever was but moderate; the wound over the femoral artery seemed disposed to unite by the first intention; that over the popliteal nearly so; that over the aneurism was suppurating. Four days after the operation, however, there was a considerable increase of the fever; erysipelas developed itself in the wounds, and gradually spread nearly over the whole surface of the limb. During the next few days the aneurismal sac sloughed out; local gangrene developed itself in a strip of skin, about one inch and a half in width, and extending from the wound over the popliteal artery, on the outside of the leg downward to about its middle. Under the use of tincture of chloride of iron internally, and the external application of pyroligneous acid, the erysipelas gradually subsided, and the sloughs detached themselves, leaving a healthy granulating surface. The ligature around the popliteal artery came away on the eleventh day, that around the femoral on the fifteenth day. Nine weeks after the operation the wounds had healed kindly; the patient had rapidly recovered his strength under the use of tonics and a nutritious diet, and had the perfect use of his limb.

An interesting point in the case is, that when under the influence of chloroform, during the period of excitation, the left arm and leg, which were completely paralyzed and useless, exhibited more strength than the sound ones. From the date of the operation the limbs gradually regained their functions, and the patient could very readily use the hand and foot when he was dismissed. The paralysis had existed for nine years.

IV. *Serous Cyst of the Neck, cured by Palpation.*—J. C., a male child, two months old, was brought to the clinic in June, 1859, on account of a congenital tumor on the left side of the neck. The child had been born with atresia of the anus, for which it had been operated upon; it was otherwise well developed and healthy. At its birth the tumor was about as large as a duck's egg, but had somewhat increased in size since that time, and extended now from the lower margin of the inferior maxillary bone to a short distance above the clavicle. It was free from pain, did not interfere with respiration or deglutition, and did not move on swallowing. The skin covering it was perfectly natural. Examined by transmitted light, the tumor was found to be to some degree transparent; it felt equally tense to the touch in all its parts, and fluctuated under pressure.

These symptoms plainly indicated the character of the swelling; it was evidently a *simple serous cyst* of the neck—the *hydrocele of the neck* of some writers. With a view to the radical cure of the affection, Professor Gross proposed the introduction of a seton; but the mother refusing to subject the child to this measure, the operation of tapping was resorted to. The tumor was punctured with a delicate trocar, the opening being followed by at least two ounces of serous fluid. The tumor disappeared, and the wound soon healed. In the early part of September, about twelve weeks after the operation, the child was brought back to the clinic, on account of the swelling having reappeared. The tumor was subjected on this occasion to a thorough examination by palpation, but no operation was performed. In the evening of the same day the patient was seized with fever and restlessness, and the swelling, increased considerably in bulk, became very painful. Three days afterwards, when the child was again seen, the tumor was found to be hot, hard, very tender on pressure —the skin covering it being discolored—and the fever having reached a high degree.

The palpation made at the previous examination had evidently excited inflammation in the cyst, and the plan of treating the case by the seton was therefore abandoned. Taking the cicatrice left after the former operation as a guide, a puncture was made in the tumor, which was followed by the escape of sero-purulent fluid, tinged with blood. Fomentations, containing acetate of lead and opium, were ordered, the patient purged with castor-oil, and one drop of tincture of aconite administered every hour until the fever had subsided. Next day the tumor was painted over with the diluted tincture of iodine, and covered with a poultice. Two days afterwards a second puncture was made, and a tent introduced into the opening. The discharge of healthy pus continued, and the fever lessened in degree. When the patient was taken home, ten days after his admission, the tumor was much diminished in size, solid, and almost free from pain; the discoloration around it had almost entirely disappeared, and the cure was nearly complete. Four weeks after the operation the child was entirely well.

V. *Ganglions of the Wrist cured by Subcutaneous Division, followed by Compression.*—Henry H., seventeen years old, by profession a barber, presented himself at the clinic of the college, in November, 1859, on account of two small, ovoidal tumors on the extensor side of the wrist, which interfered somewhat with the motion of the joint, and the exercise of his profession.

The tumors, which had began to form about seven months ago, were of a round shape, about the size of a marble, painless, unaccompanied by any discoloration of the skin, elastic, and movable. The patient could not remember having sprained or bruised the part at any time previous to their appearance. Professor Gross diagnosticated them to be *ganglions;* that is, small encysted tumors formed in the course of one of the extensor tendons.

In the *treatment* of the case a delicate tenotome was introduced subcutaneously, and the cysts cut up as minutely as possible. On puncturing the tumors, a yellowish, jelly-like substance escaped, and they became completely effaced. After the operation compression was made with a compress and piece of coin, confined by a bandage, and the patient ordered to keep his arm at rest in an elevated position, and to restrict himself to a light diet. The local inflammation following the operation was but slight, and soon subsided. Under the external application of tincture of iodine, in conjunction with the treatment here mentioned, the cure was completed within a few days, no trace of the swellings being left.

*Remarks.*—Professor Gross is inclined to ascribe the origin of tumors of this kind to inflammatory action, having observed them most commonly in hard-working people. He assumes that they are merely sacculated expansions of the sheath of the tendons, and not a new formation, as some pathologists suppose. The sac consists of a single layer, rarely thicker than the dura mater. While a common mucous burse usually secretes a fluid like ordinary synovia, a ganglion contains a yellowish, viscid, and transparent fluid, resembling white of egg, or, as in the case reported above, jelly; in old cases the contents are sometimes more solid, and consist of masses of semi-organized lymph, or small fibroid bodies, usually of the size and shape of cucumber-seeds. In a ganglion of the hand, upon which Professor Gross operated a few years ago, he observed these bodies in a variety of singular shapes—round, oval, and spindle-shaped, with a number of prolongations; they were evidently merely masses of organized lymph, which had been originally adherent to the walls of the sac, and had become detached in consequence of the friction of the tendon against the sac. Ganglions are most liable to form on the extensor tendons of the hand or wrist, although they are occasionally met with in other parts of the body. Professor Gross operated, in 1854, before the medical class of the University of Louisville, upon a young woman who had two tumors of this kind, each of the size of a small bird's egg, upon the dorsal surface of the foot. There are cases on record—and one of them has come under Professor Gross's own observation—where a ganglion was situated over the radial artery, and was, from the pulsation communicated to it, at first supposed to be an aneurism. Ganglions can be but seldom cured by stimulating applications; although the external use of iodine, and systematic compression may, in recent cases, sometimes produce absorption, and lessen the size of the tumor, the fluid in the cyst generally accumulates again. The best and most simple plan is to make firm pressure upon the sac, so as to rupture it, and allow the contents to escape into the surrounding cellular tissue; absorption will then take place, and may be rendered more speedy by the application of iodine; after the evacuation of the cyst, a compress and bandage should be applied to the part, to promote the union of the opposite side of the cavity by adhesive inflammation. In recent cases the rupture of the sac can be effected either by pressure of the thumb, or by striking the tumor with a large book; in cases of long standing, where the wall of the cyst is thicker and firmer, it is best to make a subcutaneous puncture. If the ganglion resists these measures, it is most effectually destroyed by introducing a bistoury subcutaneously, cutting it up into small pieces, and afterwards applying compression. It has been recommended by some surgeons to treat these cysts after the manner of hydrocele, by introducing a seton, or by injecting iodine; but the success which has attended this method does not speak much in its favor. As a general rule, free incision into the tumor, or excision of it, should be avoided, as it is liable to give rise to a high degree of inflammation, the results of which might endanger the use of the joint; still there are cases on record in which extirpation has been successfully practiced, and this plan of treatment has the support of old authorities, such as Celsus and Paul of Ægineta.

VI. *Polyp of the Rectum.*—Mary H., seven years old, was brought to the clinic, in October, 1859. The patient had complained for a long time of an uneasy sensation in the rectum, and had a frequent desire to evacuate her bowels; the act of defecation was attended with considerable straining, and occasionally with some bleeding; her linen was soiled by a discharge of reddish mucus from the anus; her mother's attention became directed to these symptoms, and she discovered that a small tumor protruded from the anus, whenever the child evacuated her bowels. On examining the prolapsed mass, it was found to be a polyp, consisting of a cherry-like tumor attached by a long, slender pedicle to the mucous membrane of the rectum. It was painful on being touched, of a scarlet color, soft and spongy in its consistence, and evidently highly vascular.

*Operation.*—Preparatory to the operation, the patient was freely purged, so as to avoid disturbance of the bowels for several days afterwards. The tumor having been forced beyond the sphincter, by straining over the chamber, it was seized with a vulsellum, and a ligature applied around its pedicle, as high up as it could be reached. It sloughed off very readily, but there remained a small portion of the pedicle within the rectum, which gave rise occasionally to slight bleeding, and may require further treatment. The bleeding, however, has of late disappeared, and the general health is greatly improved.

VII. *Stone in the Bladder; Lateral Operation; Unusually Small Calculus; Cure.*—John P., twenty-eight years of age, a miner, who had recently arrived from California, was admitted to the clinic of the College, in October, 1859. He had suffered for the last eighteen months from incontinence of urine, pain in making water, which increased as soon as the bladder was emptied; frequent desire to micturate, pain and itching at the head of the penis and at the orifice of the urethra, and a sense of uneasiness in the lower part of the pelvis. His urine became cloudy as soon as it began to cool, from the admixture of a considerable quantity of mucus. Professor Gross, suspecting the presence of a stone in the bladder, examined the case by sounding. The patient, who had sufficiently recovered from the effect of his voyage, was ordered a mild laxative the evening before the examination, in order to rid the bowels of impacted fæcal matters. A steel sound of moderate diameter was introduced into the bladder, the patient being in the recumbent posture, and was distinctly felt to grate against a foreign substance on the left side of the bladder; it was at first found impossible to produce the characteristic click, but at a subsequent examination it became quite perceptible. The introduction of the finger into the rectum led to the detection of a slight enlargement of the prostate gland, but afforded otherwise only a negative result.

The existence of a stone having been fully ascertained, Professor Gross proposed to remove it by the lateral operation, to which he gives the decided preference over all other procedures, on account of the remarkable success which has hitherto attended it. The patient was subjected for a few days to a preparatory treatment, consisting of rest, abstinence from animal food, and the administration of aperient medicine.

*Operation.*—The patient having been put under the influence of chloroform, and placed in a proper posture, a grooved staff was introduced into the bladder, and confided to an assistant, with the direction to incline it slightly toward the right side, so as to make the curved portion bear against the left side of the perineum. The operator, kneeling on the right knee, introduced the left index finger into the rectum, to induce it to contract, and to ascertain the position of the staff; and stretching the skin of the perineum with the thumb and fingers of the left hand, introduced the point of a slender, sharp-pointed scalpel, about an inch and a quarter above the verge of the anus, near the raphé, on its left side, and made an incision three inches in length, carrying it obliquely downward and outward, to a point midway between the tuberosity of the ischium and the anus. Then placing the point of the left index finger in the upper angle of the wound, he divided, by repeated strokes, the deep cellular tissue of the perineum, the transverse muscle, and a portion of the triangular ligament, with a few of the fibres of the elevator muscle of the anus. The membranous portion of the urethra having thus been exposed, a little in front of the prostate gland, the operator felt for the groove of the staff, at the bottom of the wound, and having found it, cut into it through the urethra, using the finger-nail as a guide to the point of the knife. The knife, inserted into the groove of the staff through the opening in the urethra, which was about one-third of an inch in length, was now carried on into the bladder, dividing, as it passed along, the neck of the organ, and, to a limited extent, the left lobe of the prostate, in a direction obliquely downward and outward. During this step of the operation the rectum was kept out of the way by being pushed to the right side by the left index finger. The incision into the bladder was followed by a gush of urine; the knife was now removed, and the finger, lying in the bottom of the wound, placed in contact with the staff, which was then withdrawn. The finger was carried into the bladder, and, after a short search, came in contact with the stone, which was of very small size, and easily extracted with the forceps.

As soon as the stone had been removed the bladder was washed out with tepid water, so as to get rid of any fragments which might have been left behind. The hemorrhage during the operation was very slight, and easily arrested.

The *after-treatment* was conducted on the usual plan. The progress of the case was not interrupted by any bad symptom, with the exception of a slight venous hemorrhage from the bladder, which occurred four days after the operation, and was promptly arrested by the use of a silver tube, inserted through the wound into the bladder. The wound united without difficulty, and five weeks after the operation the patient was discharged, entirely well. The weight of the calculus hardly exceeded *six grains.* The severe suffering which the patient had so long endured had evidently been occasioned by the frequent intromission of the stone into the mouth of the urethra during micturition.

VIII. *Rapidly recurring Cancer of the Breast.*—This interesting case was brought before the class of the Jefferson Medical College, in November, 1859. The patient, an unmarried servant-woman, forty years of age, whose menstrual functions were performed regularly, had been operated upon for encephaloid cancer of the left breast, on five previous occasions. In the first three operations the gland was but partially removed, leaving the greater portion as the nidus for new formations. About two years and a half since the first tumor made its appearance, and was extirpated at the expiration of four months. The second soon began to develop itself, and was removed in nine months. In a few weeks the third tumor commenced to grow, and was excised in seven months.

In May, 1859, when, through the kindness of Dr. Russell, of this city, the case came under the notice of Professor Gross, a fourth tumor had made its appearance, which he removed, together with what was left of the affected gland, on the twenty-fourth of the same month, four months and a half after the last operation. It is worthy of notice that until then the tumors had never chosen the cicatrices of the former operations for their seat. After the fourth operation, however, the cicatrice became the seat of the disease; a tumor formed in the upper angle of it, and rendered a fifth operation necessary, which was performed on the seventh of September, about three months and a half after the previous operation. Since then the lower angle and middle part of the incision of last May had become involved by tumors, which, enlarging at a rapid rate, rendered interference with the knife again necessary, although but two months had elapsed since the performance of the last operation.

The tumors were removed by Professor Gross, in the presence of the class, on the fifth of November, this being the sixth operation which had been performed for the same disease in the last twenty-six months.

The woman's general health had always been excellent; she had always made good recoveries from the operations, and there is no cancerous disease in the family.

All of the tumors which had been extirpated were tuberculated, firm, and compressible, and inclosed in an imperfect cyst, formed by the condensation of the surrounding cellular tissue. There was no retraction of the nipple, and no involvement of the neighboring lymphatic glands. The subcutaneous veins were enlarged, and the tumor was the seat of sharp, shooting pains, extending to the shoulder.

Physical examination showed that the tumor was encephaloid, which was confirmed by the microscope, a great number of very large cancer cells being present.

The points of interest in this case are, the rapid recurrence of the cancer; the non-involvement of the axillary lymphatic glands; the apparent absence of cancerous cachexia; the good health of the patient; and the non-appearance of tumors at the cicatrices of the first three operations.

Excepting the rapid recurrence of the disease, this case is probably an illustration of the majority of those cases of cancer which, excluding the possibility of a mistake in the diagnosis, have been reported as cured by an operation. A patient may recover quickly from the operation, enjoy very good health, there may be no trace left of the local affection, and the disease may not recur for some time; the surgeon, believing that he has eradicated the evil, publishes his triumph, and afterwards loses sight of the case. But unless cases of cancer are watched until the death of the patient, the statistical information they afford in regard to the result of operation, are unreliable and perfectly useless.

